# Mental Health Professionals’ Technology Usage and Attitudes Toward Digital Health for Psychosis: Comparative Cross-Sectional Survey Study

**DOI:** 10.2196/68362

**Published:** 2025-03-31

**Authors:** Xiaolong Zhang, Natalie Berry, Daniela Di Basilio, Cara Richardson, Emily Eisner, Sandra Bucci

**Affiliations:** 1 Division of Psychology and Mental Health, School of Health Sciences, Faculty of Biology, Medicine and Health, Manchester Academic Health Science Centre, The University of Manchester, Oxford Road, Manchester, M13 9PL, United Kingdom, 44 161 306 0422; 2 Greater Manchester Mental Health NHS Foundation Trust, Manchester, United Kingdom; 3 Division of Health Research, Faculty of Health and Medicine, Lancaster University, Lancaster, United Kingdom

**Keywords:** digital mental health, psychosis, attitudes, implementation, smartphone app

## Abstract

**Background:**

Digital health technologies (DHTs) for psychosis have been developed and tested rapidly in recent years. However, research examining mental health professionals’ views on the use of DHTs for people with psychosis is limited. Given the increased accessibility and availability of DHTs for psychosis, an up-to-date understanding of staff perception of DHTs for psychosis is warranted.

**Objective:**

In this study, we aimed to investigate mental health professionals’ usage of technology and their perception of service users’ technology usage; their views toward the use of DHTs for psychosis, including their concerns; and barriers for implementing DHTs in a mental health setting.

**Methods:**

Two cross-sectional surveys were distributed to mental health care staff who had experience of working with individuals experiencing psychosis in the United Kingdom. Survey 1 (from April 2018 to September 2020) was distributed to 3 local UK National Health Service (NHS) trusts in Northwest England; survey 2 was administered nationally across 31 UK NHS trusts or health boards (from November 2022 to March 2024). The COVID-19 pandemic occurred between the 2 survey periods. Data were analyzed descriptively.

**Results:**

A total of 155 and 352 participants completed surveys 1 and 2, respectively. Staff reported high levels of technology ownership and usage in both surveys. In general, staff expressed positive views regarding the use of DHTs for psychosis; however, barriers and concerns, including affordability, digital literacy, and potential negative effects on service users’ mental health, were also reported. We did not find notable changes in terms of staff use of digital technology in clinical practice over time.

**Conclusions:**

Staff sampled from a broad and diverse range expressed consistent optimism about the potential implementation of DHTs in practice, though they also noted some concerns regarding safety and access. While the COVID-19 pandemic is frequently regarded as a catalyst for the adoption of digital health care tools, the sustainability of this transition from traditional to digital health care appeared to be suboptimal. To address staff concerns regarding safety and potentially facilitate the implementation of DHTs, systematic evaluation of adverse effects of using DHTs and dissemination of evidence are needed. Organizational support and training should be offered to staff to help address barriers and increase confidence in recommending and using DHTs with service users.

## Introduction

Psychosis usually occurs in late adolescence or early adulthood and can have significant personal, family, social, and economic consequences [1-3]. The duration of untreated psychosis is associated with a range of negative clinical and functional outcomes; therefore, timely access to evidence-based interventions is crucial [4-6]. Early intervention services (EIS) have been established in many countries to reduce treatment delay and improve outcomes for people who have experienced a first episode of psychosis [7,8]. Despite evidence for the effectiveness and cost-effectiveness of EIS, there are still challenges for implementing and accessing such services [9,10]. Digital health technologies (DHTs) to support people who experience psychosis have been developed and tested rapidly in recent years [11]. DHTs hold the potential to scale up mental health care and provide timely access to evidence-based intervention and management options, which could help reduce duration of untreated psychosis [12], prevent relapse [13], and facilitate self-management in near real-time [14]. A variety of digital technologies, including smartphone apps, virtual reality, and websites, have been used as tools for digital interventions and remote symptom monitoring [15,16]. Large-scale randomized controlled trials are beginning to demonstrate the efficacy of digital interventions for psychosis [17-20], moving the field on from early phase feasibility studies.

As the frontline of service provision, staff attitudes and knowledge are crucial for the successful development and implementation of DHTs [21]. Previous studies exploring staff views of digital mental have found staff were interested in using DHTs to provide care and some staff were already using digital technologies to support clinical care [22-25]. However, given the increased accessibility and availability of DHTs [26], an up-to-date understanding of staff perceptions, views and attitudes toward the use of DHTs in the context of working with people with psychosis is warranted. Furthermore, although the transition to telehealth and digital health was accelerated by the COVID-19 pandemic due to the associated social distancing restrictions [27,28], the maintained implementation of DHTs in clinical practice beyond this period is limited. For example, the 2022 European Psychiatric Association survey on digitalization found that remote mental health services were not part of standard care in half (51.43%) of the 35 European countries surveyed, and over one-third (37.14%) of the respondents reported that no legislative regulation was in place in their countries [29,30]. In addition, studies focusing on staff usage of DHTs in psychosis services postpandemic are lacking.

In this paper, we report findings from 2 surveys: survey 1 (from April 2018 to September 2020) was distributed to 3 local UK NHS (National Health Service) trusts; survey 2 was administered nationally across 31 UK NHS trusts or health boards (from November 2022 to March 2024). Our aims were to understand mental health professionals’ use of technology and their perception of service users’ technology usage, their views toward the use of DHTs for psychosis, including their concerns, and barriers for implementing DHTs in a mental health setting. Although not the primary aim of this study, we conducted a narrative exploration of the differences between staff responses from the 2 surveys to gain a deeper understanding of the implementation of DHTs in clinical practice postpandemic. A timeline of the survey recruitments and COVID-19 waves in the United Kingdom is shown in Figure S1 in Multimedia Appendix 1.

## Methods

### Participants

We surveyed mental health staff working in the UK National Health Service (NHS) in both surveys. Eligibility criteria were health care professionals working within an NHS service providing mental health support to people who experience psychosis or severe mental health problems, aged 18 years or older, and had the ability to provide informed consent. Participants not sufficiently fluent in English to complete the survey were excluded from participation. Potential participants completed screening questions in the survey to assess their eligibility. Survey 1 was disseminated in 3 mental health trusts in the Northwest of England; survey 2 was distributed in 31 NHS mental health trusts or health boards across England and Scotland (Glasgow; Edinburg).

### Data Collection

Survey 1 was developed by the research team based on findings obtained in reviewing literature and focus groups with staff working in EIS [31,32]. The survey included questions about staff perceptions of service user technology usage and engagement, and their views toward use DHTs in the context of working with and supporting psychosis service users. Survey completion time was approximately 8 minutes. Participants were asked to complete either an online or paper-based version of the survey; the online version delivered using the REDCap (Research Electronic Data Capture) [33,34] platform. Survey 1 was disseminated from April 2018 to September 2020 (ie, before and during COVID-19 pandemic wave 1).

Survey 2 was conducted from November 2022 to March 2024 (ie, post COVID-19 pandemic). The survey was adapted from survey 1 and extended to collect data about staff views on digital remote monitoring technologies (reported in a separate paper; Zhang et al, unpublished data, March 2025). Therefore, this survey was longer than survey 1 and took approximately 20‐30 minutes to complete. Demographic information, data on technology ownership rates, usage in clinical practice, and staff perspectives on the use of digital health tools were collected. Participants had the option to complete an online or paper copy of the questionnaire. The online survey was conducted using the Qualtrics (Silver Lake) platform [35]. For participants using a paper copy, a study pack with a paper survey and return postage-paid envelope was sent. Once the paper copy was returned, the answers were entered manually into the Qualtrics database. The surveys are shown in Tables S1 and S2 in Multimedia Appendix 1.

### Data Analysis

Descriptive statistics, including frequencies and percentages, and data visualization, were performed using R program (R Foundation for Statistical Computing) [36] to analyze quantitative data. The proportion of missing rates for each question were summarized and reported in the results. Free-text answers were summarized narratively using Nvivo (version 12, Lumivero) [37]. As we recruited different cohorts across the 2 surveys (ie, a regional and national sample) and used slightly different questionnaire versions (ie, survey 2 was adapted and updated from survey 1), no inferential statistical analysis was performed to determine the statistical significance of the differences between the 2 surveys.

### Ethical Considerations

The participant information sheet with the study information and consent form were embedded in the survey. Data were anonymized. Survey 1 was approved by the West of Scotland Research Ethics Committee 4 (17/WS/0221), and survey 2 was approved by the Northwest-Greater Manchester West Research Ethics Committee (22/NW/0246). Participants had the chance to enter a £20 (approximately US $25) prize draw to thank them for their time.

## Results

### Participant Characteristics

A total of 155 and 352 participants completed survey 1 and 2, respectively. A summary of participant demographic characteristics is presented in Table 1. In survey 1, median participant age was 39 (IQR 32‐47) years. The median time (years) working in current role was 3 (IQR 1‐8) years. Most participants were female (119/155, 76.77%), White (137/155, 88.39%), and had completed postgraduate level education (106/155, 68.39%). Overall, 40% (62/155) of participants were working in EIS. Participant job roles included mental health nurse (32/155, 20.65%), care coordinator (24/155, 15.48%), and psychologist (21/155, 13.55%).

**Table 1.** Characteristics of survey participants.

Characteristics	Survey 1 (n=155)	Survey 2 (n=352)
Age, median (IQR)	39 (32‐47)	40 (31‐50)
Gender, n (%)		
Women	119 (76.77)	244 (69.32)
Men	36 (23.23)	102 (28.98)
Nonbinary or third gender	—^a^	1 (0.28)
Prefer not to say or unsure	—	5 (1.42)
Ethnicity, n (%)		
Asian or Asian British	11 (7.1)	45 (12.78)
Black, Black British, Caribbean or African	3 (1.94)	20 (5.68)
White	137 (88.39)	276 (78.4)
Mixed or multiple ethnic groups	2 (1.29)	5 (1.42)
Other ethnic group	1 (0.64)	4 (1.13)
Missing	1 (0.64)	2 (0.57)
Service, n (%)		
Charity sector	7 (4.52)	—
Community mental health team	10 (6.45)	119 (33.81)
Early intervention service	62 (40)	82 (23.3)
General practice	9 (5.8)	—
Home treatment team	41 (26.45)	8 (2.27)
Inpatient unit	6 (3.87)	63 (17.9)
Secondary care psychological services	13 (8.39)	—
Assertive outreach team	—	3 (0.85)
Other	—	77 (21.88)
Missing	7 (4.52)	—
Education, n (%)		
High school	3 (1.94)	7 (1.99)
Diploma or equivalent	—	41 (11.65)
Trade, technical, or vocational training	—	6 (1.7)
College	4 (2.58)	—
Some university	5 (3.22)	—
University	36 (23.22)	129 (36.65)
Postgrad	106 (68.39)	165 (46.87)
Prefer not to say	—	1 (0.28)
Missing	1 (0.65)	—
Job title, n (%)		
Care coordinator	24 (15.48)	—
Support worker	10 (6.45)	37 (10.51)
Social worker	13 (8.39)	13 (3.69)
Mental health nurse	32 (20.65)	99 (28.12)
Psychotherapist	12 (7.74)	11 (3.12)
Psychologist	21 (13.55)	54 (15.34)
Psychiatrist	11 (7.1)	50 (14.2)
General practitioner	1 (0.64)	—
Allied health professional	7 (4.52)	28 (7.95)
Student nurse, student social worker, medical student, or student allied health professional	—	7 (1.99)
Prefer not to say	—	9 (2.56)
Other	23 (14.84)	43 (12.22)
Missing	1 (0.64)	1 (0.28)
Years working in current role, median (IQR)	3 (1–8)	4 (1.5‐10)
Regions, n (%)		
England		
Northeast	—	5 (1.42)
Northwest	155 (100)	68 (19.32)
Yorkshire and the Humber	—	55 (15.63)
East Midlands	—	34 (9.66)
West Midlands	—	2 (0.57)
East of England	—	69 (19.6)
London	—	16 (4.55)
Southeast	—	47 (13.35)
Southwest	—	40 (11.36)
Scotland	—	1 (0.28)
Missing	—	15 (4.26)

a Not available.

Participants from survey 2 had a median age of 40 (IQR 31‐50) years and a median time working of 4 (IQR 1.5‐10) years in their current role. Most responders were female (244/352, 69.32%) and White (276/352, 78.41%). Around a third held a university bachelor’s degree (129/352, 36.65%), worked in a community mental health team (119/352, 33.81%), and practiced as a mental health nurse (99/352, 28.12%).

### Staff Technology Usage

As shown in Table 2, technology usage rates among survey 1 participants were high, with almost all participants reporting using the internet (147/155, 94.84%) and a personal smartphone (135/155, 87.1%) in their daily life. Wearable technology use was less common, with less than a quarter of respondents reporting using a fitness tracker (36/155, 23.23%) and only a few using a smartwatch (18/155, 11.61%). In the work environment, most respondents indicated using a desktop computer (110/155, 70.97%) and mobile phone (102/155, 65.81%). Smartphones were used by 56.77% (88/155) participants in a work context. Tablet computers were used less compared with other types of technologies; only one third of respondents reported using a tablet in the context of their clinical work (51/155, 32.9%).

**Table 2.** Staff technology usage rates (survey 1).

Technology	Yes, n (%)	No, n (%)	Missing, n (%)
Personal use			
Internet	147 (94.84)	1 (0.65)	7 (4.52)
Smartphone	135 (87.10)	10 (6.45)	10 (6.45)
Social media	123 (79.35)	21 (13.55)	11 (7.10)
Mobile phone	116 (74.84)	24 (15.48)	15 (9.68)
Laptop computer	100 (64.52)	38 (24.52)	17 (10.97)
Tablet computer	76 (49.03)	58 (37.42)	21 (13.55)
Fitness tracker	36 (23.23)	93 (60.00)	26 (16.77)
Desktop computer	36 (23.23)	93 (60.00)	26 (16.77)
Smartwatch	18 (11.61)	109 (70.32)	28 (18.06)
Professional use			
Desktop computer	110 (70.97)	28 (18.06)	17 (10.97)
Mobile phone	102 (65.81)	41 (26.45)	12 (7.74)
Laptop computer	92 (59.35)	45 (29.03)	18 (11.61)
Smartphone	88 (56.77)	50 (32.26)	17 (10.97)
Tablet computer	51 (32.90)	85 (54.84)	19 (12.26)

In survey 2, as shown in (Figure S2 in Multimedia Appendix 1), more than half respondents used a smartphone in their clinical practice (184/352, 52.27%) with a fairly even split of Android (101/352, 28.7%) and iPhone users (94/352, 26.7%). Only 20.17% (71/352) and 7.39% (26/352) of participants respectively used tablets and wearable devices in their clinical work. Notably, nearly 40% (140/352) of participants indicated not using any digital technology in the context of their clinical practice.

Participants’ responses in survey 1 about their attitudes toward digital technologies both in general and in the context of their clinical work are presented in Table 3. Generally, participants reported having a positive attitude toward digital technologies both from a personal perspective and in the context of their clinical work in EIS. Specifically, over half of the responders endorsed “agree” or “strongly agree” to the statement they are “enthusiastic about digital devices” (86/155, 55.48%), and most participants reported that technology could play a positive role in mental health services in the future (134/155, 86.45%). Only a few participants (28/155, 18.71%) expressed negative attitudes toward digital devices in general, reporting that technology is frustrating for them.

**Table 3.** Participants’ attitudes toward digital technologies (survey 1; n=155).

Statement	Strongly disagree, n (%)	Disagree, n (%)	Neutral, n (%)	Agree, n (%)	Strongly agree, n (%)	Missing, n (%)
I am enthusiastic about electronics and digital devices	5 (3.23)	11 (7.1)	50 (32.26)	57 (36.77)	29 (18.71)	3 (1.94)
I frequently look for new software or apps	12 (7.74)	43 (27.74)	43 (27.74)	43 (27.74)	11 (7.1)	3 (1.94)
My friends would describe me as “into” the latest technology	40 (25.81)	57 (36.77)	33 (21.29)	17 (10.97)	5 (3.23)	3 (1.94)
Technology could play a positive role in mental health services in the future	1 (0.65)	3 (1.94)	14 (9.03)	101 (65.16)	33 (21.29)	3 (1.94)
For me, technology is frustrating	17 (10.97)	60 (38.71)	46 (29.68)	23 (14.84)	6 (3.87)	3 (1.94)

### Staff Perception of Service Users’ Technology Use

**Figure 1.**
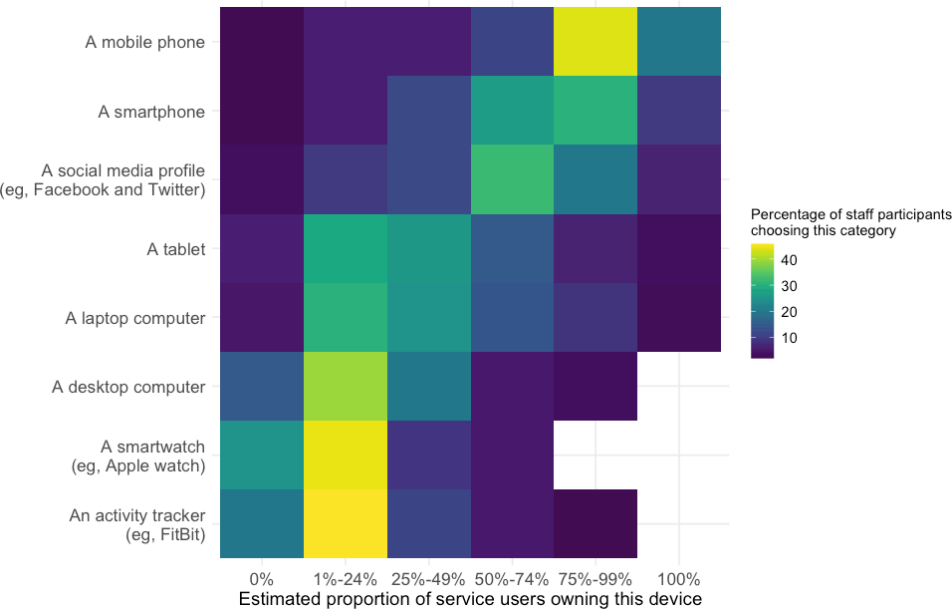
Staff estimates of service user technology types (survey 1). Yellow squares indicate that nearly half of staff chose this category, whereas dark blue squares indicate that <10% chose it.

We asked survey 1 participants to estimate service users’ technology ownership rate in percentile based on their clinical experience (Figure 1). Most staff respondents estimated that the percentage of the service users they work with who owned a mobile phone or smartphone ranged between 75%‐99%. In contrast, most staff responders estimated that the percentage of the service users they work with who owned a smartwatch and an activity tracker fell between 1%‐24%.

In survey 2, around 70% of participants (254/352, 72.1%) estimated 25%‐50% of service users on their caseload would use DHTs to support their mental health (Figure S3 in Multimedia Appendix 1). Furthermore, over half of participants (186/352, 52.8%) reported that at least 1 service user on their caseload was currently using DHTs. Of those using DHTs, staff said that service users they were working with mostly used smartphones (155/186, 83.3%) to support their mental health, followed by wearable devices (65/186, 35%) and tablets (57/186, 30.6%).

In survey 1, staff perceived barriers for service users with psychosis to own or use a mobile phone are shown in Table 4. Primary barriers were “struggling to afford phones or smartphones” (112/155, 72.26%) and “paranoia or suspiciousness about mobile phones” (101/155, 65.16%). Four additional barriers were identified from free-text responses: “struggling to afford internet, data, or Wi-Fi,” “risk of selling phones,“ “willingness to use phones,” and “restriction of accessing mobile phones in ward or prison.” Survey 2 participants reported the barriers for service users they work with to use DHTs in the free-text question, including “inability to own or use a digital device,” “paranoid beliefs about digital devices,” and “being older.“ A full list is shown in Table S3 in Multimedia Appendix 1.

**Table 4.** Staff reported barriers for service users with psychosis to own or use a mobile phone (survey 1.

Barrier	Yes, n (%)	No, n (%)	Missing, n (%)
Struggling to afford mobile phones or smartphones	112 (72.26)	33 (21.29)	10 (6.45)
Paranoia or suspiciousness about mobile phones	101 (65.16)	44 (28.39)	10 (6.45)
Damage of mobile phones	86 (55.48)	59 (38.06)	10 (6.45)
Loss of mobile phones	81 (52.26)	64 (41.29)	10 (6.45)
Technology use skills	74 (47.74)	71 (45.81)	10 (6.45)

### Using Digital Health Technologies in Clinical Practice

We asked survey 1 participants to indicate their usage of common resources, both digital and nondigital forms, in their appointments with service users (Table S4 in Multimedia Appendix 1). Many staff reported they had recommended digital resources to service users they worked with, such as online written information (120/155, 77.42%) or videos (85/155, 54.84%) about mental health. Paper-based approaches were also popular amongst staff, with 72.9% (113/155) of respondents having used paper-based symptom monitoring recorded by service users and 59.35% (92/155) having used paper-based between session therapy homework sheets.

As shown in Table S5 in Multimedia Appendix 1, participants frequently recommended digital technologies for self-management purposes, with the top recommendations including “listening to music or audio files via a smartphone to distract from voices or intrusive thoughts” (129/155, 83.23%), “using a calendar or set alarms or reminders for appointments” (127/155, 81.94%), and “setting alarms or reminders to help with medication management” (114/155, 73.55%). In contrast, symptom monitoring by a paper diary (126/155, 81.29%) was more popular than digital approaches to tracking symptoms (smartphone app: 45/155, 29.03%; website: 22/155, 14.19%). Nonetheless, most respondents were at the very least considering recommending digital symptom monitoring to service users. For example, only about 30% (45/155, 29.03%) of participants indicated they had recommended using a smartphone app to track symptoms, while around 60% (95/155, 61.29%) said they were considering recommending it. Regarding staff preferences for receiving the data from patient-generated symptom reporting through apps, half of the staff (78/155, 50.32%) wanted service users to take the data to appointments, and nearly a third (42/155, 27.1%) accepted the automatic transfer of data from apps to the care team. However, 12.26% (19/155) of respondents indicated they do not want to receive such information at all. When asked whether staff had ever recommended an app to a service user for their mental health, 79.35% (123/155) respondents said they had, whereas 43.87% (68/155) staff had recommended at least 1 app for service users to use for their physical health.

Table S6 in Multimedia Appendix 1 shows staff perceptions of the potential advantages of mental health apps. The most endorsed advantage was the “opportunity to increase understanding about symptoms and experiences” (127/155, 81.93%), followed by “opportunity for service users to take up-to-date records of symptoms and experiences to clinicians” (123/155, 79.36%), and “ability to access an app at any time and in any location” (123/155, 79.36%). Regarding barriers to staff recommending using a mental health app to service users (Table S7 in Multimedia Appendix 1), the foremost barrier reported was “service users feeling suspicious or paranoid about using smartphones and apps” (99/155, 63.87%).

We asked survey 2 participants whether they considered smartphones, tablets, and wearable devices to be useful additions for supporting their clinical practice (Figure S4 in Multimedia Appendix 1); the top rated digital devices were iPad (175/352, 49.7%), wearable devices (141/352, 40.1%), Android tablet (117/352, 33.2%), and iPhone (117/352, 33.2%). Among the 11 common features afforded by DHT to support service users, the top features rated by staff based on their perceived usefulness (all options were by more than half of respondents) were the “ability to get appointment reminders” (335/352, 95.2%), “monitoring sleep” (308/352, 87.5%) and “physical activity” (284/352 80.7%), the “ability to track mental health symptoms” (278/352, 79%), and using a “diary function” (to log thoughts and feelings (277/352, 78.7%; Table 5). Staff said that the most useful apps for service users’ mental health care were mental health apps (323/352, 91.8%), sleeping apps (322/352, 91.5%), and mindfulness apps (314/352, 89.2%; Figure S5 in Multimedia Appendix 1).

**Table 5.** Features of digital technology that participants considered useful or beneficial for service users (survey 2; n=352).

Items	Ranking	Participants, n (%)
Ability to get appointment reminders	1	335 (95.2)
Monitor sleep	2	308 (87.5)
Monitor physical activity	3	284 (80.7)
Ability to track mental health symptoms	4	278 (79.)
Diary function (ability to log thoughts and feelings in a diary)	5	277 (78.7)
Access to information/education/support	6	263 (74.7)
Ability to get information about their mental health problem	7	259 (73.6)
Ability to discuss data logged by an app with their clinician	8	246 (69.9)
Ability to self-manage	8	246 (69.9)
Ability to complete outcome measures remotely	9	237 (67.3)
Ability to share data logged by an app with a trusted friend/carer/relative for support	10	215 (61.1)
Monitor other health-related activities	11	198 (56.2)
Other	—^a^	29 (8.24)
Digital technology does not offer any benefits to service users	—	5 (1.42)

a Not available.

## Discussion

### Principal Findings

We describe the findings of 2 surveys to explore staff usage of and views on implementing DHTs in clinical practice. Survey 1 recruited a regional sample based in the northwest of England; Survey 2 reached a national sample across the United Kingdom. Both surveys recruited a sample with a median age of around 40 years old, more females than other genders, more White than other ethnicity groups, and more practiced as a nurse or a psychologist than other mental health professionals. However, in survey 2, there was a larger proportion of participants working at a community mental health team, and a smaller proportion achieved a postgraduate level of education compared with survey 1.

Staff reported fairly high levels of use of digital technologies in a clinical context in both surveys, with more than half of participants reporting using a smartphone in the context of their clinical practice (Survey 1: 56.77%; Survey 2: 52.27%). Furthermore, a significant proportion of staff expressed positive attitudes toward using digital technologies in mental health care delivery and identified themselves as generally enthusiastic about technology in the Survey 1 (55.48%). Given that staff attitudes toward DHTs and their digital technology literacy have been shown to influence the adoption of DHTs in clinical practice [38,39], our finding of largely positive attitudes suggests that staff could become key points of contact and ambassadors for DHT implementation within EIS pathways. This is also consistent with findings from previous qualitative studies, which suggest that the more clinicians understand how to use technology and feel comfortable using it, the more likely they will be to use it in clinical practice [40].

Of note, in both surveys, staff members’ estimates of service user ownership rates varied depending on device type. Specifically, staff estimated that wearable device ownership was substantially lower than smartphone ownership amongst service users with early psychosis (survey 1: smartphone 75%‐99% vs wearables 1%‐24%; survey 2: smartphone 83.3% vs wearables 35%). A similar pattern was observed in previous research on staff members’ own digital technology ownership conducted in other countries (eg, Australia [22] and China [25]). Bell et al [22] reported that clinicians who provided youth mental health care in Australia had higher usage rates of video chat and smartphones compared with wearables, social media, or virtual reality. Zhang et al [25] found that mental health staff in China were less aware of virtual reality and artificial intelligence–based interventions than social media, smartphone app, or internet-based interventions. These findings suggest that different types of digital technologies may require different implementation strategies. As the field progresses toward integrating various technologies (eg, passive sensing and smartphones for time-sensitive interventions), a lack of awareness and access to these “newer” technologies may hinder adoption. Therefore, implementation strategies might need to include providing devices such as fitness trackers [41].

Barriers and concerns to implementing DHTs were captured in both surveys and were consistent with the broader literature on barriers to implementation of DHTs, including symptoms of psychosis preventing engagement with DHTs, service user lack of motivation to use DHTs, and digital poverty [42]. Participants from both surveys expressed concerns that using such technologies could contribute to potential negative effects on service users’ mental health (eg, increased paranoia, suspiciousness, depression, or anxiety). These concerns may be related to the association between problematic use of digital technologies and mental health [43,44]. Although adverse events related to paranoia are generally rare [11], some clinical trials on digital mental health interventions for psychosis have reported adverse events in participants who experienced distress related to the technology used [45,46]. These findings highlight the urgent need for systematic evaluations and reporting of adverse effects of DHTs to fully understand their safety [47,48].

Although, as mentioned above, the usage of DHTs in clinical practice was not extensive, both surveys showed some DHT implementations in clinical practice. Many respondents from survey 1 reported using digital approaches to support the delivery of mental and physical health care support to service users. This included online videos (54.84%), written information (77.42%), and recommending relevant apps, such as those for tracking symptoms through smartphone apps (29.03%). Participants from survey 2 indicated that appointment reminders (95.2%), and monitoring sleep (87.5%), physical activities (80.7%) and mental health symptoms (79%) would be the most useful features of DHTs for mental health care. These findings are in line with survey studies conducted with staff working with young people [22] and individuals diagnosed with bipolar disorder [23], highlighting that mental health practitioners are interested in using DHTs in clinical care across various mental health settings.

The COVID-19 pandemic is often considered an accelerator for the adoption of digital health care [27] , with increased technology use in clinical practice during social distancing restrictions (eg, appointments through video calls) [49,50]. By comparing the findings of the 2 surveys, we found that this shift might have not been maintained among staff working in services that support service users with psychosis. For example, smartphones and tablets were reported to be used by 56.77% and 32.9% of participants in clinical practice in survey 1, respectively, but no increase was observed in survey 2 (smartphone 52.27% and tablet 20.17%). This echoes findings from recent reviews, which indicated that although staff across the globe had adopted telehealth during the pandemic, feedback and willingness to continue postpandemic have been mixed; the rates of telehealth use declined once restrictions loosened [51,52]. One explanation might be that the pandemic only resulted in staff using remote care temporarily to manage social distancing restrictions but did not form a “new norm” for integrating DHTs in their clinical practice [53]. In addition, lack of organizational support, such as financial support and necessary infrastructure to support digital tools, may also contribute to the drop in rates of use postpandemic [54,55]. This finding underscores that certain barriers may have persisted despite the widespread transition to telehealth and digital health. Continuous efforts are required to further understand the barriers and facilitators for implementing DHTs in psychosis services in the post-pandemic era.

### Strengths and Limitations

One strength of this study is that survey 2 reached a national sample, enabling us to gain a broader understanding beyond the local sample captured in Survey 1. In addition, by comparing and contrasting the 2 survey results, it brings insights on the potential impact COVID-19 pandemic had on staff use of, and views about, DHTs for psychosis care. There are some limitations. First, the online surveys might have attracted participants who are more familiar with and supportive of digital technologies compared with their counterparts. Second, both surveys recruited a convenience sample. As such, we cannot guarantee that participants are fully representative of all staff working in secondary care mental health settings. Third, the 2 surveys recruited different samples and used different measurements, precluding statistical comparison of the results.

### Conclusion

This study found that most staff working with individuals experiencing psychosis were familiar with digital technology, both personally and professionally. They expressed positive attitudes toward implementing DHTs in practice. However, concerns regarding the safety of using DHTs were reported. To further develop and implement DHTs, a systematic evaluation of their adverse effects is needed to address safety concerns. In addition, providing comprehensive training is essential to ensure that staff are well-equipped to adopt evidence-based tools to support service users with psychosis and to enhance their confidence in using these technologies. Furthermore, it is imperative to address organizational barriers, such as funding and IT infrastructure, in the postpandemic era, despite the acceleration of adoption of DHTs during the COVID-19 pandemic. Furthermore, it is crucial to conduct further studies with service users directly to explore the readiness, acceptability and adoption of digital health interventions as part of their health care journey.

## Supplementary material

10.2196/68362 Multimedia Appendix 1 Supplementary figures and tables.

## Abbreviations

DHT: digital health technology
EIS: early intervention services
NHS: National Health Service
NIHR: National Institute for Health and Care Research
REDCap: Research Electronic Data Capture
