# Supplementary material for: Mental Health Professionals’ Technology Usage and Attitudes Toward Digital Health for Psychosis: Comparative Cross-Sectional Survey Study
Source: JMIR Ment Health. 2025 Mar 31;12:e68362. doi: 10.2196/68362 (PMC11975120; doi:10.2196/68362)
Supplement: Multimedia Appendix 1 [file mental-v12-e68362-s001.docx]

**Supplementary Figure 1.** Timeline of the survey recruitments and COVID-19 waves in the UK

**Note:** The blue arrows show the timeline, with the grey boxes above indicating the recruitment period of each survey and the yellow boxes below indicating the time when the two COVID-19 waves occurred in the UK. The exact time is labelled at the beginning and the end of each time box.

**Supplementary Figure 2**. Staff digital technology usage as part of clinical practice (survey 2)

**Supplementary Figure** **3**. Staff estimated percentage of caseload that would use DHTs to support mental health (survey 2).

**Supplementary** **Figure 4**. Staff perceived usefulness of different types of digital technologies as an addition to support clinical practice (survey 2).

**Supplementary** **Figure 5**. Types of apps that staff considered useful for service users’ mental health care (Survey 2).

**
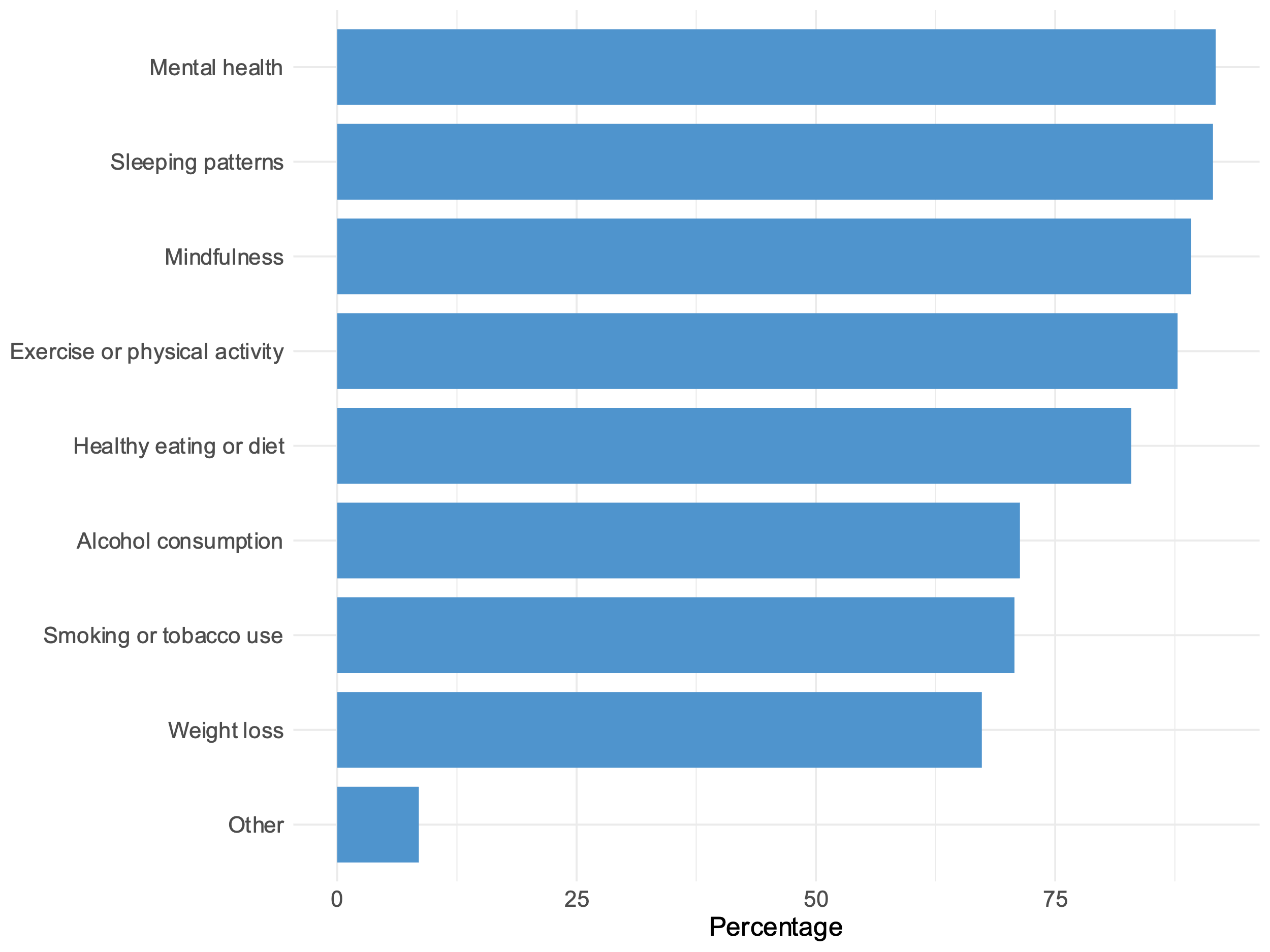
**

**Supplementary Table 1.** Phase 1 survey

**Demographic information**

1. How old are you? ……..

2. What is your gender? ……………………

3. What is your ethnicity? ……………………

4. What service do you currently work in?

Early Intervention Service Community Mental Health Team

Home Treatment Team Secondary care psychological services

Charity sector Inpatient unit

General Practice

5. What is the highest level of education you have attended? Please tick to indicate

High school/Secondary school University (degree awarded)

College/Sixth form Postgraduate course

Some University

(degree not or not yet awarded)

6. What is your job title?

Care coordinator Support worker Social worker

Community Psychiatric Nurse Occupational therapist Psychotherapist

Psychologist Psychiatrist GP

Other - please state …………………………..

7. How long have you been working in your current role? (in years) ……………………………..

8. Have you ever referred a client to a research trial? (please tick to indicate)

Yes No

9. What factors would influence your decision to refer clients to a research trial? (please tick all that apply)

Evidence-base Professional past experience

Client risk Clients current prescription

Client preference Type of research

Clinical guidelines Resources/availability

MDT opinions Other (please state) …………………………….

**Technology ownership/use questions**

10. Which of the following do you use? (please circle to indicate):

| Mobile phone (personal) | Yes | No |
| --- | --- | --- |
| Mobile phone (work) | Yes | No |
| Smartphone (personal) | Yes | No |
| Smartphone (work) | Yes | No |
| Tablet computer (e.g. Ipad) (personal) | Yes | No |
| Tablet computer (e.g. Ipad) (work) | Yes | No |
| Laptop computer (personal) | Yes | No |
| Laptop computer (work) | Yes | No |
| Desktop computer (personal) | Yes | No |
| Desktop computer (work) | Yes | No |
| Internet | Yes | No |
| Social media | Yes | No |
| Smartwatch (e.g. Apple watch) | Yes | No |
| Fitness tracker (e.g. Fitbit) | Yes | No |

11. Please indicate how much you agree or disagree with the following statements? (please circle):

| Question | Strongly disagree | Disagree | Neither agree nor disagree | Agree | Strongly agree |
| --- | --- | --- | --- | --- | --- |
| I am enthusiastic about electronics and digital devices | 1 | 2 | 3 | 4 | 5 |
| I frequently look for new software or apps | 1 | 2 | 3 | 4 | 5 |
| My friends would describe me as “into” the latest technology | 1 | 2 | 3 | 4 | 5 |
| Technology could play a positive role in mental health services in the future | 1 | 2 | 3 | 4 | 5 |
| For me, technology is frustrating | 1 | 2 | 3 | 4 | 5 |

**Service users’ technology use/ownership**

12. Please provide an **estimate** of the proportion of your current caseload who own…: (please circle to indicate)

| A mobile phone | 0% | 1-24% | 25-49% | 50-74% | 75-99% | 100% |
| --- | --- | --- | --- | --- | --- | --- |
| A smartphone | 0% | 1-24% | 25-49% | 50-74% | 75-99% | 100% |
| A social media profile (e.g. Facebook, Twitter) | 0% | 1-24% | 25-49% | 50-74% | 75-99% | 100% |
| A tablet | 0% | 1-24% | 25-49% | 50-74% | 75-99% | 100% |
| A laptop computer | 0% | 1-24% | 25-49% | 50-74% | 75-99% | 100% |
| A desktop computer | 0% | 1-24% | 25-49% | 50-74% | 75-99% | 100% |
| A smartwatch (e.g. Apple Watch) | 0% | 1-24% | 25-49% | 50-74% | 75-99% | 100% |
| An activity tracker (e.g. FitBit) | 0% | 1-24% | 25-49% | 50-74% | 75-99% | 100% |

13. What barriers have you experienced service users with psychosis facing, if any, in being able to own or use a mobile phone? (please tick all that apply):

Struggling to afford mobile phones/smartphones Loss of mobile phones

Damage of mobile phones Technology use skills

Paranoia/suspiciousness about mobile phones Other (please state below)

Not applicable (I have not encountered any barriers) ………………………………..

14. Have you ever recommended an app to a service user to help with their physical health (e.g. stop smoking app, weight management app)? (please tick to indicate)

Yes No

15. Have you ever recommended an app to a service user to help with their mental health (e.g. mindfulness)

Yes No

16. Have you ever had the following experiences of communicating with service users via technology? (please circle to indicate)

| Text messages to offer practical support (e.g. appointment, visit or medication reminders) | Yes | No |
| --- | --- | --- |
| Text messages to offer emotional support (e.g. coping strategies, encouraging comments) | Yes | No |
| Emails to offer practical support (e.g. appointment, visit or medication reminders) | Yes | No |
| Emails to offer emotional support (e.g. coping strategies, encouraging comments) | Yes | No |
| Checking service users’ social media profiles to see how they are getting on | Yes | No |
| Accepting friend requests from service users via social media | Yes | No |
| Rejecting friend requests from service users via social media | Yes | No |

17. Are you aware of any Trust guidelines surrounding the following topics?

a. Communicating with service users via text messages:

Yes No Unsure

b. Communicating with service users using personal mobile phone numbers:

Yes No Unsure

c. Viewing service users’ social media profiles:

Yes No Unsure

d. Accepting friend requests from service users on social media:

Yes No Unsure

18. Have you ever recommended the following strategies to service users for self-management? (please circle to indicate)

| Tracking symptoms/experiences via a smartphone app | Yes | No (but I would consider recommending in the future) | No (and I wouldn’t consider recommending in the future) |
| --- | --- | --- | --- |
| Tracking symptoms/experiences via a website | Yes | No (but I would consider recommending in the future) | No (and I wouldn’t consider recommending in the future) |
| Tracking symptoms/experiences using a paper diary | Yes | No (but I would consider recommending in the future) | No (and I wouldn’t consider recommending in the future) |
| Listening to music or audio files via a smartphone to distract from voices or intrusive thoughts | Yes | No (but I would consider recommending in the future) | No (and I wouldn’t consider recommending in the future) |
| Recording auditory hallucinations via a smartphone | Yes | No (but I would consider recommending in the future) | No (and I wouldn’t consider recommending in the future) |
| Photographing visual hallucinations via a smartphone | Yes | No (but I would consider recommending in the future) | No (and I wouldn’t consider recommending in the future) |
| Setting alarms/reminders to help with medication management | Yes | No (but I would consider recommending in the future) | No (and I wouldn’t consider recommending in the future) |
| Using a calendar or set alarms/reminders for appointments | Yes | No (but I would consider recommending in the future) | No (and I wouldn’t consider recommending in the future) |
| Accessing an online forum to access support/connect with others | Yes | No (but I would consider recommending in the future) | No (and I wouldn’t consider recommending in the future) |

19. Have you ever used any of the following resources during appointments with service users? (please circle to indicate)

| Online written information about mental health | Yes | No (but I would consider doing this in the future) | No (and I wouldn’t consider doing this in the future) |
| --- | --- | --- | --- |
| Online videos (e.g. on YouTube) about mental health | Yes | No (but I would consider doing this in the future) | No (and I wouldn’t consider doing this in the future) |
| Smartphone camera to visually record formulations  Not applicable to my role | Yes | No (but I would consider doing this in the future) | No (and I wouldn’t consider doing this in the future) |
| Symptom monitoring information recorded by service users via a smartphone | Yes | No (but I would consider doing this in the future) | No (and I wouldn’t consider doing this in the future) |
| Symptom monitoring information recorded by service users via a website | Yes | No (but I would consider doing this in the future) | No (and I wouldn’t consider doing this in the future) |
| Symptom monitoring information recorded by service users using a paper diary | Yes | No (but I would consider doing this in the future) | No (and I wouldn’t consider doing this in the future) |
| A smartphone app to complete therapy tasks between sessions  Not applicable to my role | Yes | No (but I would consider doing this in the future) | No (and I wouldn’t consider doing this in the future) |
| A website to complete therapy tasks between sessions  Not applicable to my role | Yes | No (but I would consider doing this in the future) | No (and I wouldn’t consider doing this in the future) |
| Paper-based therapy tasks between sessions  Not applicable to my role | Yes | No (but I would consider doing this in the future) | No (and I wouldn’t consider doing this in the future) |

20. To what extent do you agree or disagree with the following statements? (please circle to indicate)

| Question | Strongly disagree | Disagree | Neutral | Agree | Strongly agree |
| --- | --- | --- | --- | --- | --- |
| Social media contributes towards depression and/or anxiety | 1 | 2 | 3 | 4 | 5 |
| Social media contributes towards paranoia or suspiciousness | 1 | 2 | 3 | 4 | 5 |
| Social media can make voices worse | 1 | 2 | 3 | 4 | 5 |
| Social media can help service users interact with friends and/or family | 1 | 2 | 3 | 4 | 5 |
| Using social media can help service users socialise with people | 1 | 2 | 3 | 4 | 5 |
| Using social media makes people compare themselves with others | 1 | 2 | 3 | 4 | 5 |
| It would be beneficial for service users to engage in a social media group with others with psychosis | 1 | 2 | 3 | 4 | 5 |

21. Would you want to receive the symptom information (e.g. anxiety, depression, hallucinations) service users have entered into a mobile phone app? (please tick to indicate)

Yes (I would want the information to be automatically transferred to the team)

Yes (I want to receive the information, but I would want service users to take it to appointments themselves to show me, rather than have data automatically transferred)

No (I would not want to see the symptom information service users enter on an app)

22. If you were told about a new trial implementing a smartphone app informed by the principles of CBT to help people with psychosis self-manage their mental health, do you think you would refer service users to receive it? (please tick to indicate)

Yes No Unsure

Please could you provide the reason(s) for the answer you provided above:

………………………………………………………………………………………………………………………………………………………………………………………………………………………………………………………………………………………………………………………………………………………………………

23. To what extent do you agree or disagree (1 = strongly disagree; 5 = strongly agree) that the following barriers would affect the likelihood of you referring service users to receive a mental health app? (please tick all that apply)

| Barrier | Strongly disagree | Disagree | Neutral | Agree | Strongly agree |
| --- | --- | --- | --- | --- | --- |
| Smartphone handset and data costs | 1 | 2 | 3 | 4 | 5 |
| Service users’ technology and/or literacy skills | 1 | 2 | 3 | 4 | 5 |
| My own technology skills and knowledge |  |  |  |  |  |
| Poor motivation to engage with an app | 1 | 2 | 3 | 4 | 5 |
| Concerns about how helpful a mental health app would be | 1 | 2 | 3 | 4 | 5 |
| Concerns a mental health app would be used as an excuse to replace face-to-face support | 1 | 2 | 3 | 4 | 5 |
| Privacy/confidentiality of data inputted | 1 | 2 | 3 | 4 | 5 |
| Service users feeling suspicious or paranoid about using smartphones and apps | 1 | 2 | 3 | 4 | 5 |
| Concerns about identifying risk | 1 | 2 | 3 | 4 | 5 |
| Apps will miss the therapeutic relationship | 1 | 2 | 3 | 4 | 5 |

24. Please list any other concerns that you can think of that would prevent you from referring a service user to a trial providing a CBT-informed smartphone app.

………………………………………………………………………………………………………………………………………………………………………………………………………………………………………………

25. To what extent do you agree or disagree (1 = strongly disagree; 5 = strongly agree) that the following are potential advantages of mental health apps? (please tick all that apply)

|  | Strongly disagree | Disagree | Neutral | Agree | Strongly agree |
| --- | --- | --- | --- | --- | --- |
| Ability to access an app at any time and in any location | 1 | 2 | 3 | 4 | 5 |
| Opportunity to take control over mental health needs | 1 | 2 | 3 | 4 | 5 |
| Opportunity to increase understanding about symptoms and experiences | 1 | 2 | 3 | 4 | 5 |
| Apps can be anonymous | 1 | 2 | 3 | 4 | 5 |
| Opportunity for service users to take up-to-date records of symptoms and experiences to clinicians | 1 | 2 | 3 | 4 | 5 |
| Opportunity to identify symptom triggers and patterns | 1 | 2 | 3 | 4 | 5 |
| Cost-effective alternative to face-to-face support | 1 | 2 | 3 | 4 | 5 |
| Potential for staff to intervene early if increases in symptoms are identified | 1 | 2 | 3 | 4 | 5 |
| Increased privacy in comparison to paper-based monitoring or tasks | 1 | 2 | 3 | 4 | 5 |
| Having a mental health app available is normalising and de-stigmatising | 1 | 2 | 3 | 4 | 5 |

26. Please list any other advantages of using mental health apps

………………………………………………………………………………………………………………………………………………………………………………………………………………………………………………………………………………………………………………………………………………………………………

**Supplementary Table 2.** Phase 2 survey

Part 1: Demographics

| 1. ***Please select your gender***   Do you identify as:   - Man / Male - Woman / Female - Non-binary/third gender - Prefer not to say/unsure - Other (please state):  ………………………….   ***Does your gender match with your sex assigned at birth?***   - Yes - No - Prefer not to say | 1. ***How old are you?***     …………………………………………………………. |
| --- | --- |
| 1. ***What is your ethnic origin?***   **White**   - English, Welsh, Scottish, Northern Irish or British - Irish - Gypsy or Irish Traveller - Roma - Any other White background   **Asian or Asian British**   - Indian - Pakistani - Bangladeshi - Chinese - Any other Asian background   **Black, Black British, Caribbean or African**   - Caribbean - African - Any other Black, Black British, or Caribbean background   **Mixed or multiple ethnic groups**   - White and Black Caribbean - White and Black African - White and Asian - Any other Mixed or multiple ethnic background   **Other ethnic group**   - Arab - Any other ethnic group - Prefer not to say | 1. ***What is your highest completed level of education?***  - Primary school - Secondary school - Diploma or equivalent - Trade/technical/vocational training - University bachelor’s degree - University master’s degree - PhD or higher - Prefer not to say |
| 1. ***What is your job title?***  - Mental health nurse - Social worker - Support worker / nursing assistant / STR worker / healthcare assistant - Psychotherapist (or trainee) - Psychiatrist (consultant, trainee, non-training grade) - Psychologist (qualified, assistant, trainee) - Allied health professional (please state) …………………………. - Student nurse / student social worker / medical student / student allied health professional - Prefer not to say - Other (please state)  ……………………………………………. | 1. ***How long have you worked in your current role (in years)?***     **…...........................................** |

Part 2: Technology Use Questions

What are Digital Health Tools?

In this survey we are asking about apps, sensing features on a smartphone, wearable devices or smartwatches that are used for things like monitoring symptoms, counting steps, helping with sleep routine, and setting medication reminders. We are interested in how these devices are used for things like sleep, mental health tracking, heart rhythm or location (whereabouts).

Here are some pictures of different digital health tools:


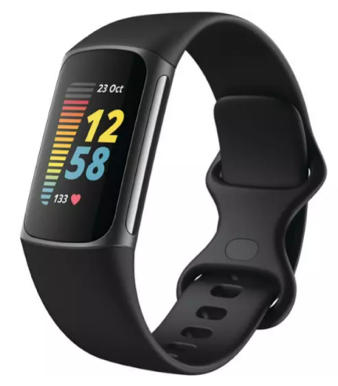

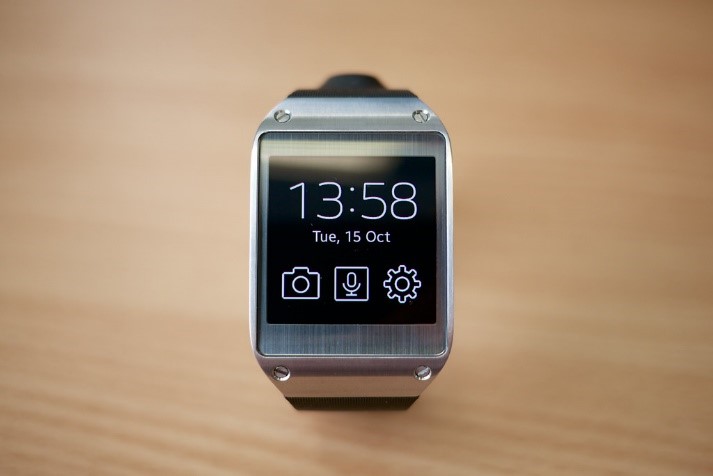


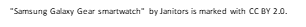


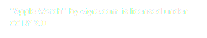


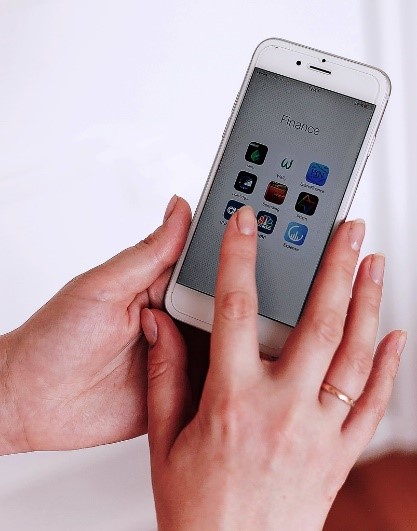


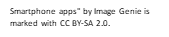


1. Do you currently use any digital technologies as part of your clinical practice (i.e. direct work with service users)?

- Smartphone – iPhone
- Smartphone – Android
- Smartphone – Other
- Tablet – iPad
- Tablet – Android
- Tablet – Other
- Wearable device/smartwatch
- None
- Other. Please specify ……………………………..

1. Which of the following digital technologies do you think would be a useful addition to support your clinical practice? (Please select all that apply)

- Smartphone – iPhone
- Smartphone – Android
- Smartphone – Other
- Tablet – iPad
- Tablet – Android
- Tablet – Other
- Wearable device/smartwatch
- Other. Please specify ……………………………..
- None – digital technologies would not support my practice

1. What percentage of service users on your case load do you think would use digital health tools (e.g. smartphone apps, wearable devices/smartwatches) to support their mental health?

|  |  |  |  |  |
| --- | --- | --- | --- | --- |
| 0% | 25% | 50% | 75% | 100% |
| 1. Are any service users you work with using digital health tools currently to support their mental health?  - Yes    If yes… What are they using?   - Smartphone - Tablet - Wearable device/smartwatch - Other. Please specify …………………………….. | | | | |
| 1. If 0% to 75%...   Please tell us why you think some of the service users you work with would not use digital health tools.  ……………………………………………………………………………………………………………………………………………  …………………………………………………………………………………………………………………………………………… | | | | |
| - No If no, skip to Q10. | | | | |

1. What percentage of service users on your caseload do you think would benefit from digital health tools (e.g. smartphone apps, wearable devices/smartwatches) to support their mental health (including those who already use them)?

|  |  |  |  |  |
| --- | --- | --- | --- | --- |
| 0% | 25% | 50% | 75% | 100% |
| 1. If 0% to 75%...   Please tell us why you think some service users you work with would not benefit from digital health tools  ……………………………………………………………………………………………………………………………………………  …………………………………………………………………………………………………………………………………………… | | | | |

1. What features of digital technology do you think would be useful or beneficial for service users? (please select all that apply)

- Ability to get appointment reminders
- Ability to get information about their mental health problem
- Ability to complete outcome measures remotely
- Ability to track mental health symptoms
- Monitor sleep
- Monitor physical activity
- Monitor other health-related activities
- Ability to self-manage
- Diary function (ability to log thoughts and feelings in a diary)
- Ability to share data logged by an app with a trusted friend/carer/relative for support
- Ability to discuss data logged by an app with their clinician
- Access to information/education/support
- Other. Please state ………………………
- Digital technology does not offer any benefits to service users

1. What types of apps do you think would be useful for service users to use as part of their mental health care? (Please select all that apply)

- Mental health
- Exercise or physical activity
- Healthy eating or diet
- Smoking or tobacco use
- Sleeping patterns
- Alcohol consumption
- Weight loss
- Mindfulness
- Other Please specify ……………………….

Part 3: Implementing Digital Health Tools into Clinical Practice

1. What training or infrastructure would you need to use digital health tools (e.g. smartphone apps, wearable devices/smartwatches) in your clinical practice? (Select all that apply):

- Regular training (updates)
- One off training session
- Funding
- Support from the clinical team
- Support from management
- Ongoing technical support (regarding using the device)
- Someone specifically employed to support staff and service users in how to use digital devices and interpret data flows (A ‘Digital Navigator’)

1. Is there any other training or infrastructure you would need/want?

…………………………………………….………………………………………………………………………………...

1. Would you like to see digital health tools (e.g. smartphone apps, wearable devices/smartwatches) implemented/used in your service?

- Yes
- No
- Not Sure
- Other ……………………………………………………………………………………………………………….

1. If yes…

Why? ………………………………………………………………………………………………………………………….

What tools would you like to see implemented? ……………………………………………………….

1. If No/Not sure

Why? ………………………………………………………………………………………………………………………….

1. Please indicate how much you agree or disagree with the following statements about digital health tools (e.g. smartphone apps, wearable devices/smartwatches).

| **Question** | **Strongly disagree** | **Disagree** | **Neither agree nor disagree** | **Agree** | **Strongly agree** |
| --- | --- | --- | --- | --- | --- |
| I think digital health tools would be effective in supporting a service user’s mental healthcare | 1 | 2 | 3 | 4 | 5 |
| I think digital health tools would be safe in a mental health setting | 1 | 2 | 3 | 4 | 5 |
| I think my service could support the long-term use of digital health tools | 1 | 2 | 3 | 4 | 5 |
| I think using digital health tools in clinical practice would help/improve my workload | 1 | 2 | 3 | 4 | 5 |
| I think using digital health tools in clinical practice would help me deliver better care to service users | 1 | 2 | 3 | 4 | 5 |
| I think using digital health tools in clinical practice would be concerning/worrisome because of data privacy or storage of personal data | 1 | 2 | 3 | 4 | 5 |
| I think using digital health tools in clinical practice would be difficult for staff who are not familiar with these devices/technologies | 1 | 2 | 3 | 4 | 5 |
| I think using digital health tools in clinical practice would be a burden on staff | 1 | 2 | 3 | 4 | 5 |
| I think using digital health tools in clinical practice would be costly | 1 | 2 | 3 | 4 | 5 |
| I think using digital health tools in clinical practice would be time-consuming | 1 | 2 | 3 | 4 | 5 |
| I think using digital health tools in clinical practice could take the focus away from other needs of the service (money or time better spent elsewhere) | 1 | 2 | 3 | 4 | 5 |
| I think some service users will be further marginalised because they don’t have money/skills to use digital health tools | 1 | 2 | 3 | 4 |  |

1. If you would like to add any further information/explanation of your answers, please do so here.

……………………………………………………………………………………………………………………………………………………………………………………………………………………………………………………………………………………………….

1. What functions would you like to see digital health tools used for in clinical practice (Please tick to indicate)?

| **Question** | **What digital health tool would you like to be used here?** | | | |
| --- | --- | --- | --- | --- |
|  | **Smartphone App** | **Wearable Device** | **Neither** | **Other. Please state** |
| I would like a digital health tool to alert/remind service users about healthcare appointments | ​​☒​ | ​​☐​ | ​​☐​ | ……………… |
| I would like a digital health tool to alert/remind service users to take their medication | ​​☐​ | ​​☐​ | ​​☐​ | ……………… |
| I would like a digital health tool to prompt service users to record how they are feeling | ​​☐​ | ​​☐​ | ​​☐​ | ……………… |
| I would like a digital health tool to remind service users to complete homework (between session)/at home activities | ​​☐​ | ​​☐​ | ​​☐​ | ……………… |

The next few questions are about smartphone apps.

1. To what extent do you agree or disagree (1 = strongly disagree; 5 = strongly agree) that the following are potential advantages of smartphone apps for mental healthcare? (Please select all that apply)

| **Question** | **Strongly disagree** | **Disagree** | **Neutral** | **Agree** | **Strongly agree** |
| --- | --- | --- | --- | --- | --- |
| Health-related apps can help service users take control over their mental health | 1 | 2 | 3 | 4 | 5 |
| Apps allow for more honest and valid data to be collected from a service user compared to administering questionnaires or asking service users directly about their symptoms | 1 | 2 | 3 | 4 | 5 |
| Apps provide service users the opportunity to record and reflect on symptoms and experiences over time | 1 | 2 | 3 | 4 | 5 |
| Apps provide the opportunity for service users to share information in ‘real-time’ with their clinical team | 1 | 2 | 3 | 4 | 5 |
| Apps provide the opportunity for service users to identify triggers and patterns | 1 | 2 | 3 | 4 | 5 |
| Service users may feel that information in an app is more private compared to talking to a member of their clinical team | 1 | 2 | 3 | 4 | 5 |
| Apps might help reduce health inequalities by enabling access to services in new ways | 1 | 2 | 3 | 4 | 5 |

1. Please list any other reasons why, in your opinion, service users might want to use an app to manage their mental health.

………………………………………………………………………………………………………………………………………………………………………………………………………………………………………………………………………………………………

1. Do you have any ideas for any other ways that an app could help service users manage their mental health?

………………………………………………………………………………………………………………………………………………………………………………………………………………………………………………………………………………………………

1. Do you have any ideas for ways in which apps can help support your clinical practice / ways of working with service users?

………………………………………………………………………………………………………………………………………………………………………………………………………………………………………………………………………………………………

The next few questions are about wearable devices (e.g. Apple Watch or Fitbit)

1. To what extent do you agree or disagree (1 = strongly disagree; 5 = strongly agree) that the following are potential advantages of wearable devices in mental healthcare? (Please select all that apply)

| **Question** | **Strongly disagree** | **Disagree** | **Neutral** | **Agree** | **Strongly agree** |
| --- | --- | --- | --- | --- | --- |
| Wearable devices can help service users take control over their mental health | 1 | 2 | 3 | 4 | 5 |
| Wearable devices allow for more honest and valid data to be collected from a service user compared to administering measures or asking service users directly about their symptoms | 1 | 2 | 3 | 4 | 5 |
| Wearable devices provide service users the opportunity to record and reflect on symptoms and experiences over time | 1 | 2 | 3 | 4 | 5 |
| Wearable devices provide service users the opportunity to share information in ‘real-time’ with their clinical team | 1 | 2 | 3 | 4 | 5 |
| Wearable devices provide service users the opportunity to identify triggers and patterns | 1 | 2 | 3 | 4 | 5 |
| Service users may feel that information collected by a wearable device is more private compared to talking to a member of their clinical team | 1 | 2 | 3 | 4 | 5 |
| Wearable devices might help reduce health inequalities by enabling access to services in new ways | 1 | 2 | 3 | 4 | 5 |

1. Please list any other reasons why, in your opinion, service users might want to use a wearable device to manage their mental health.

………………………………………………………………………………………………………………………………………………………………………………………………………………………………………………………………………………………………

1. Do you have any ideas for any other ways that a wearable device could help service users manage their mental health?

………………………………………………………………………………………………………………………………………………………………………………………………………………………………………………………………………………………………

1. Do you have any ideas for ways in which wearable devices can help support your clinical practice / ways of working with service users?

………………………………………………………………………………………………………………………………………………………………………………………………………………………………………………………………………………………………

**Supplementary Table 3.** Reasons for service users would not use digital health technologies (DHTs) for mental healthcare (Survey 2)

| **Coding** | **Number of coding references** |
| --- | --- |
| Inability to own or use a digital device | 74 |
| Paranoid beliefs about digital devices | 44 |
| Older age | 35 |
| Cost | 25 |
| Too unwell to use a digital device | 24 |
| Lack of motivation to use a digital device | 20 |
| Chaotic lifestyle | 12 |
| Risk of losing, selling, or damaging devices | 5 |
| Not seeing the benefit | 5 |
| Tracking may provoke anxiety | 5 |
| Cognitive impairment | 4 |
| Lack of engagement | 4 |
| Not aware of DHTs | 4 |
| Mistrust or dislike of technology | 3 |
| Worried about data security | 3 |
| Prefer to continue with existing treatment | 2 |
| Lack of insight | 2 |
| Practical issues | 2 |
| Staff lack of skill to implement DHTs | 2 |
| Able to use technology but not for health purposes | 1 |
| Cultural resistance | 1 |
| DHTs seen as minimising or diminishing their problems | 1 |
| Ineffectiveness | 1 |
| Lack of clinical guidelines to support the use of DHTs | 1 |
| Service users prefer face-to-face care | 1 |
| Worried about risks of using technology | 1 |

**Supplementary Table 4.** Resources used during appointments with service users (Survey 1, n=155): N (%)

| **Resources** | **Yes** | **No, but would consider in future** | **No, but would not consider in future** | **Not Applicable** | **Missing** |
| --- | --- | --- | --- | --- | --- |
| Online written information about mental health | 120 (77.42) | 15 (9.68) | 2 (1.29) | 4 (2.58) | 14 (9.03) |
| Online videos (e.g. on YouTube) about mental health | 85 (54.84) | 44 (28.39) | 8 (5.16) | 5 (3.23) | 13 (8.39) |
| Smartphone camera to visually record formulations | 24 (15.48) | 58 (37.42) | 24 (15.48) | 36 (23.23) | 13 (8.39) |
| Symptom monitoring information recorded by service users via a smartphone | 32 (20.65) | 90 (58.06) | 13 (8.39) | 6 (3.87) | 14 (9.03) |
| Symptom monitoring information recorded by service users via a website | 11 (7.1) | 101 (65.16) | 22 (14.19) | 7 (4.52) | 14 (9.03) |
| Symptom monitoring information recorded by service users using a paper diary | 113 (72.9) | 19 (12.26) | 4 (2.58) | 6 (3.87) | 13 (8.39) |
| A smartphone app to complete therapy tasks between sessions | 23 (14.84) | 71 (45.81) | 11 (7.1) | 35 (22.58) | 15 (9.68) |
| A website to complete therapy tasks between sessions | 20 (12.9) | 73 (47.1) | 12 (7.74) | 35 (22.58) | 15 (9.68) |
| Paper-based therapy tasks between sessions | 92 (59.35) | 12 (7.74) | 3 (1.94) | 32 (20.65) | 16 (10.32) |

**Supplementary Table 5.** Strategies recommended to service users for self-management (Survey 1, n=155): N (%)

| **Strategies** | **Yes** | **No, but consider recommending** | **No, would not consider recommending** | **Missing** |
| --- | --- | --- | --- | --- |
| Tracking symptoms/experiences via a smartphone app | 45 (29.03) | 95 (61.29) | 4 (2.58) | 11 (7.1) |
| Tracking symptoms/experiences via a website | 22 (14.19) | 107 (69.03) | 14 (9.03) | 12 (7.74) |
| Tracking symptoms/experiences using a paper diary | 126 (81.29) | 14 (9.03) | 4 (2.58) | 11 (7.1) |
| Listening to music or audio files via a smartphone to distract from voices or intrusive thoughts | 129 (83.23) | 10 (6.45) | 3 (1.94) | 13 (8.39) |
| Recording auditory hallucinations via a smartphone | 16 (10.32) | 91 (58.71) | 37 (23.87) | 11 (7.1) |
| Photographing visual hallucinations via a smartphone | 8 (5.16) | 85 (54.84) | 50 (32.26) | 12 (7.74) |
| Setting alarms/reminders to help with medication management | 114 (73.55) | 24 (15.48) | 6 (3.87) | 11 (7.1) |
| Using a calendar or set alarms/reminders for appointments | 127 (81.94) | 14 (9.03) | 3 (1.94) | 11 (7.1) |
| Accessing an online forum to access support/connect with others | 95 (61.29) | 42 (27.1) | 7 (4.52) | 11 (7.1) |

**Supplementary Table 6.** Staff views on potential advantages of mental health apps (Survey 1, n=155): N(%)

| **Advantages** | **Strongly disagree** | **Disagree** | **Neutral** | **Agree** | **Strongly agree** | **NA** |
| --- | --- | --- | --- | --- | --- | --- |
| Ability to access an app at any time and in any location | 1 (0.65) | 4 (2.58) | 9 (5.81) | 75 (48.39) | 48 (30.97) | 18 (11.61) |
| Opportunity to take control over mental health needs | 2 (1.29) | 6 (3.87) | 20 (12.9) | 79 (50.97) | 30 (19.35) | 18 (11.61) |
| Opportunity to increase understanding about symptoms and experiences | – | 4 (2.58) | 7 (4.52) | 90 (58.06) | 37 (23.87) | 17 (10.97) |
| Apps can be anonymous | – | 8 (5.16) | 28 (18.06) | 80 (51.61) | 22 (14.19) | 17 (10.97) |
| Opportunity for service users to take up-to-date records of symptoms and experiences to clinicians | – | 3 (1.94) | 12 (7.74) | 89 (57.42) | 34 (21.94) | 17 (10.97) |
| Opportunity to identify symptom triggers and patterns | – | 2 (1.29) | 11 (7.1) | 82 (52.9) | 43 (27.74) | 17 (10.97) |
| Cost-effective alternative to face-to-face support | 21 (13.55) | 40 (25.81) | 38 (24.52) | 29 (18.71) | 10 (6.45) | 17 (10.97) |
| Potential for staff to intervene early if increases in symptoms are identified | 2 (1.29) | 9 (5.81) | 28 (18.06) | 82 (52.9) | 17 (10.97) | 17 (10.97) |
| Increased privacy in comparison to paper-based monitoring or tasks | 3 (1.94) | 21 (13.55) | 57 (36.77) | 44 (28.39) | 13 (8.39) | 17 (10.97) |
| Having a mental health app available is normalising and de-stigmatising | 2 (1.29) | 7 (4.52) | 23 (14.84) | 80 (51.61) | 24 (15.48) | 19 (12.26) |

**Supplementary Table 7.** Barriers for referring service users to receive a mental health app (Survey 1, n=155): N(%)

| **Barriers** | **Strongly disagree** | **Disagree** | **Neutral** | **Agree** | **Strongly agree** | **NA** |
| --- | --- | --- | --- | --- | --- | --- |
| Smartphone handset and data costs | 1 (0.65) | 16 (10.32) | 25 (16.13) | 74 (47.74) | 21 (13.55) | 18 (11.61) |
| Service users’ technology and/or literacy skills | 1 (0.65) | 16 (10.32) | 25 (16.13) | 74 (47.74) | 21 (13.55) | 18 (11.61) |
| My own technology skills and knowledge | 31 (20) | 54 (34.84) | 22 (14.19) | 20 (12.9) | 2 (1.29) | 26 (16.77) |
| Poor motivation to engage with an app | 2 (1.29) | 18 (11.61) | 39 (25.16) | 69 (44.52) | 10 (6.45) | 17 (10.97) |
| Concerns about how helpful a mental health app would be | 6 (3.87) | 37 (23.87) | 46 (29.68) | 43 (27.74) | 6 (3.87) | 17 (10.97) |
| Concerns a mental health app would be used as an excuse to replace face-to-face support | 7 (4.52) | 38 (24.52) | 45 (29.03) | 32 (20.65) | 16 (10.32) | 17 (10.97) |
| Privacy/confidentiality of data inputted | 6 (3.87) | 24 (15.48) | 31 (20) | 54 (34.84) | 22 (14.19) | 18 (11.61) |
| Service users feeling suspicious or paranoid about using smartphones and apps | 2 (1.29) | 15 (9.68) | 22 (14.19) | 78 (50.32) | 21 (13.55) | 17 (10.97) |
| Concerns about identifying risk | 2 (1.29) | 17 (10.97) | 37 (23.87) | 60 (38.71) | 22 (14.19) | 17 (10.97) |
| Apps will miss the therapeutic relationship | 2 (1.29) | 24 (15.48) | 30 (19.35) | 52 (33.55) | 28 (18.06) | 19 (12.26) |
